# Supplementary material for: An Electrochemical Amperometric Ethylene Sensor with Solid Polymer Electrolyte Based on Ionic Liquid
Source: Sensors (Basel). 2021 Jan 21;21(3):711. doi: 10.3390/s21030711 (PMC7864481; doi:10.3390/s21030711)
Supplement: Supplementary file 1 [file sensors-21-00711-s001.pdf]

Supplementary materials

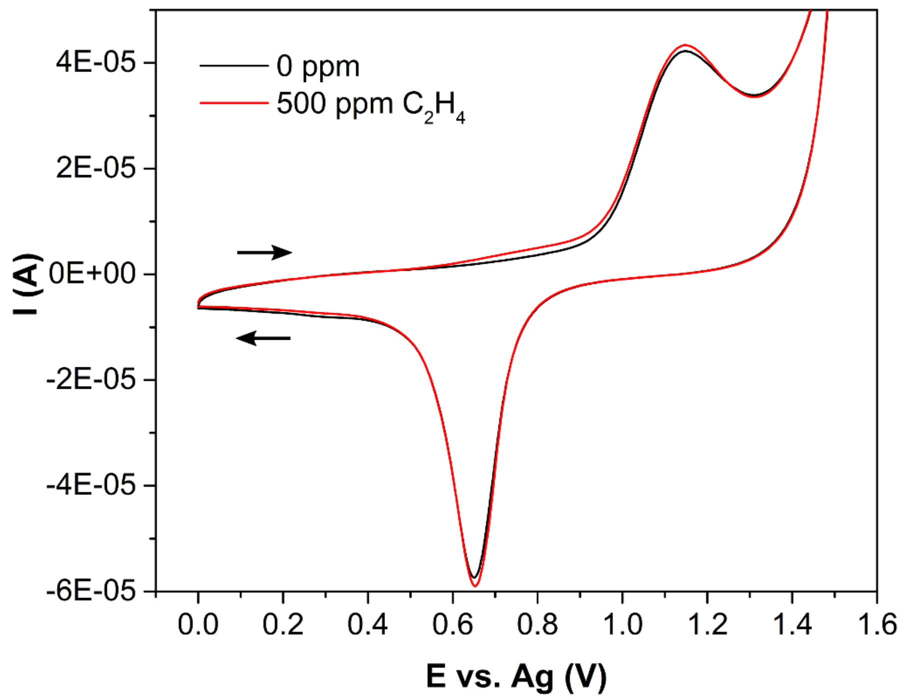

Figure S1. Current-voltage characteristics of the ionic liquid with/without gaseous ethylene (23 °C, 40 %RH, 101.325 kPa, 1 L/min, scan rate = 50 mV/s).

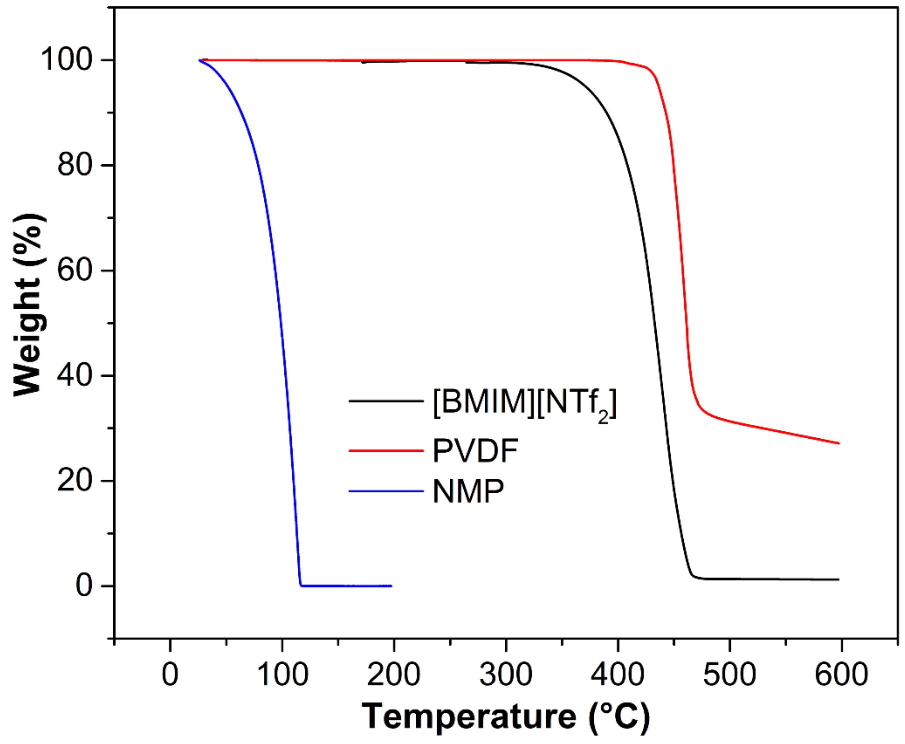

Figure S2. Thermogravimetric curves for components of the solid polymer electrolyte.
